# Prediction of Reactive Nitrous Acid Formation in Rare‐Earth MOFs via ab initio Molecular Dynamics

**DOI:** 10.1002/anie.202102956

**Published:** 2021-04-08

**Authors:** Dayton J. Vogel, Jessica M. Rimsza, Tina M. Nenoff

**Affiliations:** ^1^ Nanoscale Sciences Department Sandia National Laboratories Albuquerque NM 87185 USA; ^2^ Geochemistry Department Sandia National Laboratories Albuquerque NM 87185 USA; ^3^ Material, Physical, and Chemical Sciences Sandia National Laboratories Albuquerque NM 87185 USA

**Keywords:** ab initio calculations, adsorption, metal–organic frameworks (MOFs), nitrous acid, rare-earth metals

## Abstract

Reactive gas formation in pores of metal–organic frameworks (MOFs) is a known mechanism of framework destruction; understanding those mechanisms for future durability design is key to next generation adsorbents. Herein, an extensive set of ab initio molecular dynamics (AIMD) simulations are used for the first time to predict competitive adsorption of mixed acid gases (NO_2_ and H_2_O) and the in‐pore reaction mechanisms for a series of rare earth (RE)‐DOBDC MOFs. Spontaneous formation of nitrous acid (HONO) is identified as a result of deprotonation of the MOF organic linker, DOBDC. The unique DOBDC coordination to the metal clusters allows for proton transfer from the linker to the NO_2_ without the presence of H_2_O and may be a factor in DOBDC MOF durability. This is a previously unreported mechanisms of HONO formation in MOFs. With the presented methodology, prediction of future gas interactions in new nanoporous materials can be achieved.

## Graphical abstract

Ab initio molecular dynamics (AIMD) simulations are used to predict competitive adsorption of mixed acid gases (NO_2_ and H_2_O) and the in‐pore reaction mechanisms for a series of rare‐earth metal–organic frameworks with DOBDC linkers. For the first time, spontaneous formation of nitrous acid (HONO) is identified as a result of deprotonation of the MOF organic linker.

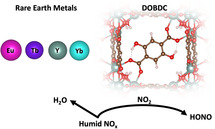

## Introduction

Chemical separations are necessary to produce everyday products and services used throughout the world and account for about 40 % of US industrial energy use.^[1]^ 80 % of worldwide energy production is still generated using fossil fuels.^[2]^ Chemical separations are needed to treat flue gas streams which can contain parts per million levels of reactive NO_*x*_ (NO_2_/NO), SO_*x*_, CO_2_ acid gases, along with humidity.[^3^, ^4^, ^5^] The presence of acid gas species in flue gas streams contribute to its toxicity. They also present challenges for separation materials since the intrinsic reactivity of acid gas species results in material poisoning and degradation of many adsorbents. Material degradation in the presence of acid gases also increases costs as current materials may only be applied once before losing separation capability.

To address these challenges, significant research has focused on a class of nanoporous materials called metal‐organic frameworks (MOFs), which have exhibited excellent qualities for chemical separations.[^6^, ^7^, ^8^, ^9^, ^10^, ^11^, ^12^] MOFs are synthesized through a judicious selection of metal cluster elements and organic linker molecules, enabling targeted framework compositions, pore size, and ligand functional groups. Furthermore, MOF materials can be tailored to contain unique material characteristics for specific chemical environments.

One method for tuning MOF material properties is to choose a metal precursor with new chemical properties. The development of MOF materials synthesized with lanthanide (Ln) and actinide elements provide unique advantages compared to traditional transition metal MOFs.[^13^, ^14^, ^15^] The inclusion of f block elements offer new electronic structures which can result in unique MOF topologies, and novel optical and magnetic properties.

Recently, a new series of rare earth 2,5‐dihydroxyterephtalic acid (RE‐DOBDC) MOFs have been synthesized with their crystal structure based on the Zr UiO‐66 framework.[^16^, ^17^] The RE‐DOBDC MOFs maintain a similar framework that is approximate to UiO‐66 but provide unique chemical and electronic properties attributed to rare earth elements. Previous DFT calculations have also shown consistency with experimentally measured IR spectra when modelled as fully activated materials, mimicking the experimental conditions.[^17^, ^18^] The unique electronic structures provided by Ln 4f electrons offer binding properties not exhibited by Zr. Utilization of the RE elements in the MOF metal centre enabled preferential binding of acid gases, such as NO_*x*_ and SO_*x*_, to these frameworks.[^19^, ^20^, ^21^] In addition to the selective adsorption of acid gases, this series of RE‐DOBDC MOFs have shown to be structurally stable under humid NO_*x*_ environments, and both optically and magnetically responsive to NO_*x*_ adsorption in humid environments.[^20^, ^21^, ^22^, ^23^] The responsiveness of the RE‐DOBDC MOFs to NO_*x*_ adsorption stems from two primary adsorption sites: i) an unsaturated metal centre and ii) the carboxylates on the DOBDC organic linkers. Resulting chemical species in the MOF pore (post‐humid NO_*x*_ exposure) were experimentally identified as nitrate, nitrite, and nitro functional groups.^[20]^

Previously published experimental work identified newly formed gas species and adsorption site competitions in RE‐DOBDC MOFs.^[20]^ However, the effect of species’ formation mechanisms or binding on MOF structural stability was not investigated. This is necessary in order to obtain atomic level insight into adsorption mechanisms and processes. Static density functional theory (DFT) simulations at 0 K were performed and validated that H_2_O is more strongly bound to the RE metal sites than NO_*x*_, which strongly interacts with the DOBDC linkers.^[20]^ While these previous DFT studies were able to shed some light on the adsorption process, several assumptions were made, including the concentration and binding location of the acid gas molecules in the structure. Furthermore, the simulations were unable to identify possible formation of secondary molecular species (both long lasting and temporary species), the effect of spatial confinement by the organic linkers on adsorption, and the role of temperature. Since MOFs are comprised of flexible organic linkers, material motion and gas‐framework interactions influence the separation capability and needs to be considered in investigations of acid gas separation. Ultimately, an understanding of the structure‐property relationship between framework composition and durability is required for the design of MOFs toward applications in more complex, caustic environments. Ab initio molecular dynamics (AIMD) simulations enable real world predictions toward that end.

AIMD combines accurate electronic structure calculations found in DFT and large scale classical molecular dynamic (MD) simulations by implementing DFT electronic structure optimizations at each time step along a MD trajectory. AIMD is computationally expensive but is offset by the level of insight into the energy and chemical behaviour of highly interacting gas‐MOF environments. This is true especially for systems containing NO_*x*_ species which contain an unpaired electron. All calculations are spin unrestricted,^[24]^ allowing for the spin to be taken into account. Previous AIMD work has been used to predict competitive adsorption and separations of O_2_ versus N_2_ in M_2_(DOBDC),^[25]^ C6 alkane isomers,^[26]^ and to describe the mechanisms of adsorption and desorption of H_2_O in M_2_(DOBDC).^[27]^ Additionally, AIMD simulations have been applied to identify unique breathing modes found in MOF materials^[28]^ and hydrogen uptake in MOF‐74.^[29]^ While these works attest to the insight and predictive capabilities of AIMD simulations, previous work has stopped short of application of AIMD to the simulations of complex multicomponent mixed acid gases.

The application of advanced computing simulations, such as AIMD, are required to address the role of how acid gas mixtures react inside the RE‐DOBDC MOF pores. Examples of this include studies of the formation of molecular species, binding energies between the acid gas species and the framework, and framework compensation for local defects. In particular, identification of chemical by‐products in situ is very difficult experimentally given the fast time scales and high number of simultaneous interactions. Using AIMD provides valuable atomistic mechanism information that cannot be achieved elsewise.

To overcome the inherent shortfalls of standard computational investigations we present, for the first time, an AIMD investigation of separation of complex mixed acid gas environments in RE‐DOBDC MOFs. To identify the mechanisms that control the interactions of mixed acid gases with RE‐DOBDC MOFs, 24 unique dynamic AIMD simulations were performed for both single gas adsorption of NO_2_ and H_2_O and mixed gas compositions of NO_2_ and H_2_O in RE‐DOBDC MOFs (RE=Eu, Tb, Y, Yb) at ambient temperatures. The suite of AIMD simulations enabled the exploration of the effect of acid gas composition and concentration on the in‐pore formation of various nitrous biproducts. These simulations enable the first computational investigation of this novel and stable RE‐DOBDC MOF series in response to complex mixed acid gas environments, with the results providing mechanistic‐based insights that can be used to predict the material response to even more complex acid gas mixtures.

Additionally, detailed analysis focuses on the interaction between the RE‐DOBDC MOF framework and NO_2_ adsorbed gas molecules. Previous experimental results had identified multiple unique reactions occurring between humid NO_*x*_ and RE‐DOBDC MOFs including interaction between NO_*x*_ and the DOBDC linker.^[20]^ NO_2_ adsorption or desorption at unsaturated RE metal sites, reactions between NO_2_–DOBDC hydroxyl linkers, C‐NO_2_ nitro formation, N_2_O_4_ formation, and HONO formation were all explored. The formation of HONO is highlighted as it is an important precursor to the hydroxyl radical, which is detrimental in atmospheric chemistry.^[30]^ Furthermore, HONO has also been shown to reactively interact with UiO‐66‐NH_2_ MOFs, resulting in HONO being broken down into N_2_ and H_2_O.^[31]^

The application of AIMD allows for the simulation of spontaneous reactions between in‐pore acid gas molecules, and between the acid gas molecules and the MOF framework. Through this complex AIMD simulation series, the preponderance of HONO formation and its bonding to MOF frameworks is highlighted. Additionally, unique chemical insights are presented from more realistic temperature affects as compared with static DFT calculations. Importantly, the use of AIMD enables the future design of novel and optimized nanoporous materials for selective adsorption and durability to more complex and industrially relevant acid gas stream.

## Results and Discussion

The application of AIMD allows for the simulation of spontaneous reactions between individual acid gas molecules inside the MOF pore, and between the acid gas molecule and the MOF framework. The results allow for the evaluation of competitive adsorption between NO_2_ and H_2_O at both the unsaturated metal centres and the DOBDC linkers. Pure and binary mixtures of H_2_O and NO_2_ were evaluated for identification of how competitive effects may impact adsorption and separation. The complete data set of acid gas adsorption for all 24 structures are included in the SI (Tables S1,S2).

### Selection of acid gas mixtures

**Figure 1:**
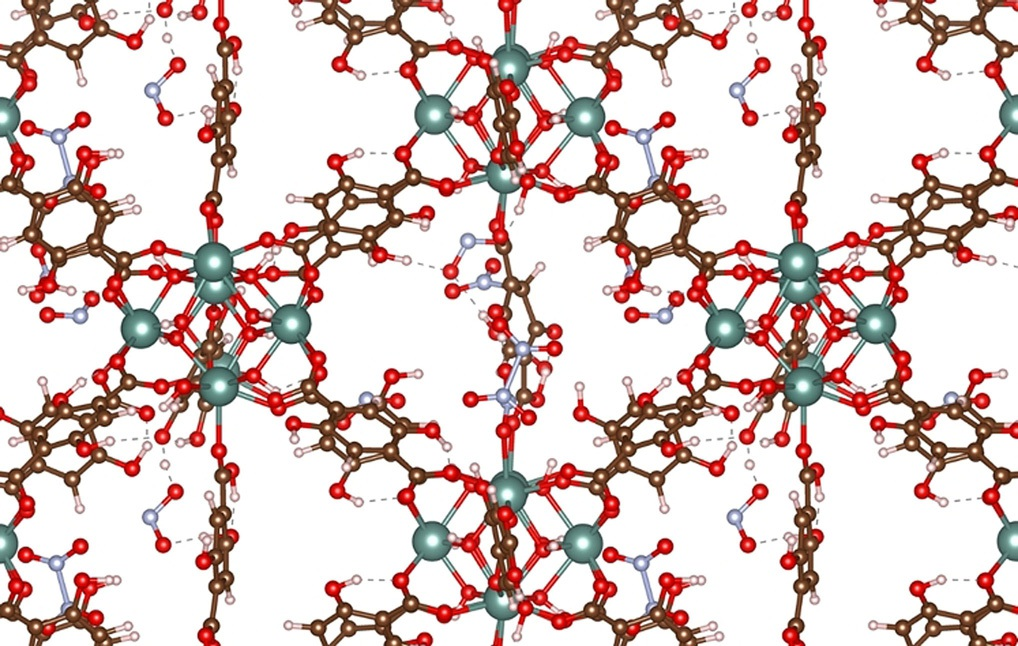
Image of Y‐DOBDC + 12 NO_2_ comprised of multiple calculation unit cells to show MOF structure and coordination. Dotted lines show H‐bonding. Y (green), O (red), C (brown), H (white), and N (blue).

A total of 24 unique AIMD trajectories were designated as vital to determining the effects of MOF metal centre choice, gas selectivity in adsorption, including gas composition, gas pressure, and adsorption temperature. First, four different metal centres (RE=Eu, Tb, Y, Yb) of the RE‐DOBDC MOF were evaluated to identify their influence on adsorption. Next, single gas compositions were simulated for H_2_O and NO_2_ as a benchmark for comparing with more complex gas mixtures. Binary gas mixtures (H_2_O:NO_2_) were then simulated to identify direct competitive effects between two different acid gases. The increase in complexity of the gas mixtures allows for the simulation of competitive effects between gas species in adsorption behaviour. The final variable was gas concentration, with both low and high concentrations of each gas mixture being simulated (i.e. 1 NO_2_ molecule versus 12 NO_2_ molecules, 1:1 H_2_O:NO_2_ versus 6:6 H_2_O:NO_2_). The ratio and composition of acid gases simulated in all AIMD trajectories are included in Table 1. The RE‐DOBDC MOF structures contain 12 metal atoms in the unit cell, therefore a maximum of 12 acid gas molecules were included in the simulation. This enabled gas adsorption at 100 % of the metal sites. The complete data set of acid gas adsorption for all 24 trajectories is included in the SI (Tables S1,S2) and an example RE‐DOBDC structure is included in Figure 1.

**Table 1:** Ratio and acid gas species for all AIMD trajectories in Eu, Tb, Y, and Yb ‐DOBDC MOFs.

Rare‐Earth Metal	Number of Gas Molecules	Gas Composition
Eu, Tb, Y, Yb	1	H_2_O or NO_2_
Eu, Tb, Y, Yb	12	H_2_O or NO_2_
Eu, Tb, Y, Yb	2	1 H_2_O:1 NO_2_
Eu, Tb, Y, Yb	12	6 H_2_O:6 NO_2_

### Metal dependence of adsorption

Overall, limited variation in adsorption of H_2_O and NO_2_ at the metal centres in the RE‐DOBDC MOFs were hypothesized, based on previously calculated DFT binding energies for H_2_O and NO_2_ in RE‐DOBDC MOFs, which identified that H_2_O is bound more strongly than NO_2_ across the entire Ln‐series.^[32]^ Additionally, the statically calculated binding energies for H_2_O and NO_2_ in RE‐DOBDC MOFs reported by Vogel et al.^[32]^ exhibited only a ≈0.1 eV variation in binding energies in RE‐DOBDC MOFs. Here, metal centre dependence of adsorption across all AIMD trajectories included 9 total adsorption events at metal centres in the Eu‐DOBDC trajectories, 4 in Tb‐DOBDC, 6 in Y‐DOBDC and 4 Yb‐DOBDC, Table S2. Along the calculated AIMD trajectories, adsorption at a RE metal site is classified as gas adsorption to a RE atom without desorption. This trend in metal adsorption events closely follows the Ln contraction trend in ionic 3+ radii Eu > Tb > Y > Yb for RE elements.^[33]^ The decreasing ionic radii size results in a decrease in unit cell size and RE‐DOBDC MOF pore volume. This places the gas molecules in closer proximity to each other in the pore and increases the number of gas‐gas and gas‐DOBDC interactions.^[24]^ Tb shows a lower number of adsorption compared to the smaller Y. However, it can be contributed to the small number of events along the trajectory. Therefore, the trend in gas adsorption indicates that it is the volume of the unit cell, Table S3, and not necessarily the chemistry of the metal centre, that is causing increased metal site adsorption through more frequent competitive interactions.

### Gas adsorption dependence on concentration

**Figure 2:**
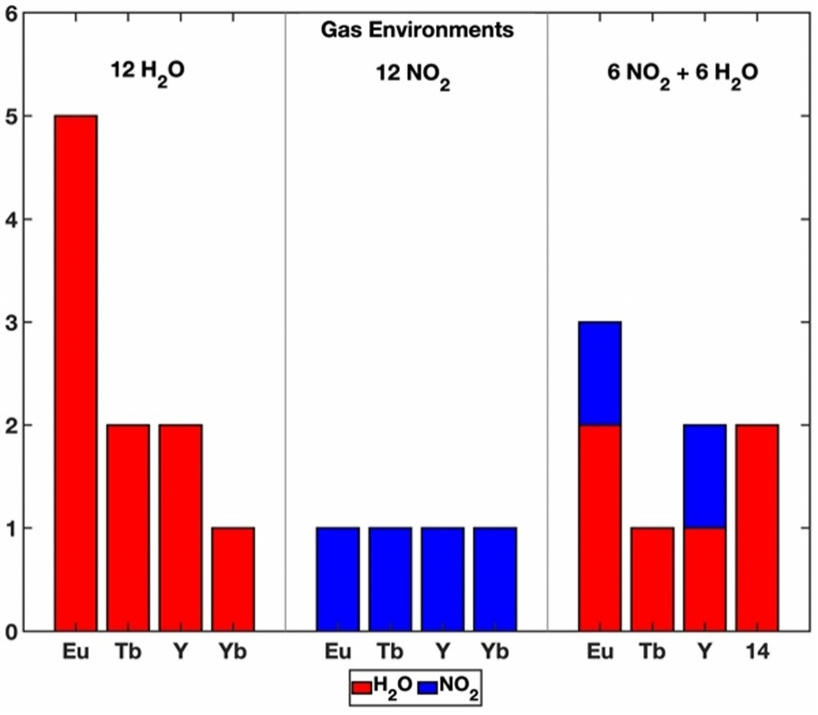
Number of gas adsorption events at unsaturated RE metal sites in RE‐DOBDC MOFs in high concentration gas environments of 12 H_2_O, 12 NO_2_, and 6 NO_2_ + 6 H_2_O.

Gas adsorption at metal sites is the primary mechanism of adsorption in MOF materials and occurs in all AIMD trajectories. When analysing the effects of gas concentration in the RE‐DOBDC MOF analogues, comparison of adsorption behaviour between low and high concentration gas mixtures shows a clear distinction. In low gas concentrations containing a single H_2_O molecule adsorption at a metal site occurs only in the Y‐DOBDC MOF. However, high concentration H_2_O gas mixtures (12 H_2_O) account for 95 % of all identified metal site adsorption events along the AIMD trajectories, Figure 2, with adsorption occurring in the Eu, Tb, Y, and Yb ‐DOBDC MOFs. At low concentrations, the individual acid gases were observed to interact with the DOBDC linkers, hindering their ability to diffuse to an unsaturated metal site, resulting in minimal adsorption. The formation of new chemical species is also concentration dependent, with increased formation of new chemical species and increased gas adsorption along the AIMD trajectories, Figure 2 and Table S1.

### Gas adsorption dependence on composition

Identification of competitive adsorption is investigated through comparisons of high concentration single and binary H_2_O and NO_2_ gas mixtures. Initially focusing on pure NO_2_ gas, limited gas‐metal adsorption in the 12 NO_2_ trajectories was observed, with a total of 4 adsorption events. The low number of adsorption events highlights the weak adsorption energy of NO_2_ at unsaturated metal sites in RE‐DOBDC MOFs compared with H_2_O or mixed H_2_O + NO_2_ gases. In comparison to pure NO_2_, the 12 H_2_O trajectories show a total of 9 adsorption events and indicates a stronger interaction of H_2_O at the metal sites. This finding is in agreement with previous static DFT calculations in RE‐DOBDC MOFs which identified a binding energy trend of H_2_O > NO_2_ across the RE‐DOBDC MOF series at unsaturated metal sites.^[32]^

NO_2_ is known to adsorb to both metal centres and ligands,^[20]^ which may be affected through competition with H_2_O adsorption. In the binary 6 NO_2_ + 6 H_2_O gas mixture, the NO_2_ adsorption is reduced compared to pure 12 NO_2_. In all mixed gas trajectories, NO_2_ is the least common molecule bound to a metal centre, accounting for only 25 % of the bound species in the 6 NO_2_ + 6 H_2_O trajectories, indicating competitive metal site adsorption between NO_2_ and H_2_O. The new competing interactions are hypothesized as NO_2_‐H_2_O gas‐gas interactions and H_2_O‐metal adsorption. However, as the number of NO_2_ adsorption events decreased following the introduction of H_2_O into the AIMD trajectories, from 4 to 2, the total number of gas adsorption events at metal centres increased, from 4 to 8. The increased metal site adsorption is due specifically to H_2_O adsorption, highlighting the stronger H_2_O interaction when directly competing with NO_2_. As the H_2_O preferentially binds at the metal sites, NO_2_ begins to adsorb in a different pore location.

Despite the energetic preference for H_2_O adsorption at the metal centre and the high adsorption of NO_2_ on the linker, metal site adsorption of NO_2_ was still identified. The AIMD trajectories highlighted a series of conditions which allow the NO_2_ to adsorb at an unsaturated metal site and are detailed later in this manuscript.

### Formation of secondary molecular species in MOFs

**Figure 3:**
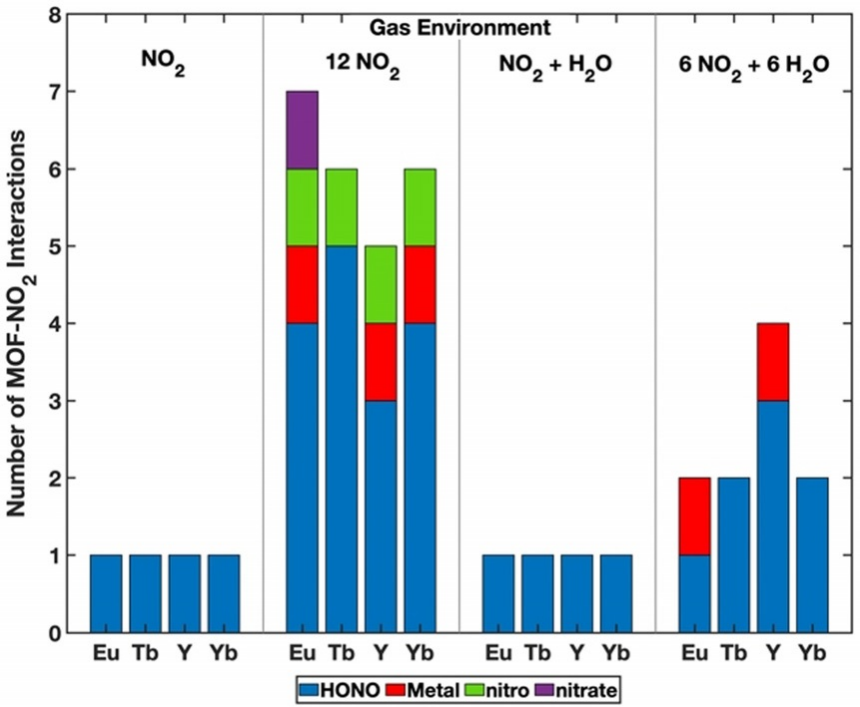
The total number of strong MOF‐NO_2_ interactions are presented for Eu, Tb, Y, and Yb ‐DOBDC in acid gas environments containing NO_2_: NO_2_, 12 NO_2_, NO_2_ + H_2_O, and 6 NO_2_ + 6 H_2_O. The number of new HONO species (blue), NO_2_ adsorbing to a metal site (red), nitrate formation (purple), and nitro formation (green) are presented for each RE element of the RE‐DOBDC MOF, in the respective gas environments.

In complex gas mixtures, newly formed by‐product species play a role in competitive gas‐MOF interactions. In studying the effects of competitive gas adsorption, the data shows five new species observed across the AIMD subset: HONO, N_2_O_4_, nitrate groups, nitro groups, and H_3_O^+^. The new individual molecular species N_2_O_4_ is formed via 2 NO_2_⇌N_2_O_4_, while H_3_O^+^ is formed via H_2_O deprotonation of a DOBDC hydroxyl, see Scheme S3. The formation mechanisms of new nitrate and nitro groups are provided in Schemes S1,S2, respectively, and the formation of HONO explored in depth later in this manuscript. The total number of MOF‐NO_2_ interactions are presented for Eu, Tb, Y, and Yb ‐DOBDC in four acid gas environments: NO_2_, 12 NO_2_, NO_2_ + H_2_O, and 6 NO_2_ + 6 H_2_O in Figure 3.

The most reactive gas species identified in the AIMD trajectories was NO_2_, as it was calculated to adsorb at metal sites and resulted in by‐product species within the RE‐DOBDC MOFs. In the data set, all of the AIMD trajectories that contained NO_2_ resulted in the formation of nitrogen based gas‐MOF interactions that were not present the beginning of AIMD trajectories, Figure 3. Adsorption data identifies 5 NO_2_ binding events at metal centres, 5 NO_2_ binding events to ligands, 32 HONO molecules formed, 4 N_2_O_4_ molecules formed, 4 nitro (R‐NO_2_) groups formed, and 1 nitrate (R‐ONO_2_) formed across the 4 RE‐DOBDC MOFs studied.

The formation of a HONO, nitro, or nitrate species in all calculated RE‐DOBDC structures indicates a strong interaction of NO_2_ with multiple parts of the MOF framework. Specifically, the formation of HONO, nitro, and nitrate all result from a NO_2_‐DOBDC linker interaction. However, following the introduction of H_2_O, the predominance of HONO is slightly reduced and the formation of nitro and nitrate groups are not seen. This indicates a change in how NO_2_ interacts with the DOBDC linkers. The changes are hypothesized to be attributed to NO_2_‐H_2_O interactions and H_2_O‐DOBDC interactions, that can interfere with HONO, nitro, and nitrate formation. The concentrations and mechanism of formation of these by‐products are discussed in detail below.

### Identification of NO_2_ interactions with the MOF

As previously stated, the strong NO_2_‐DOBDC interactions allow for the formation of new chemical species, including new N−O or N−C chemical bonds in the RE‐DOBDC pore. The formation of N−O bonds can occur at the DOBDC anchoring carbonyl O atoms or at a DOBDC hydroxyl group, as they are the most available O framework atoms. The interactions with linker O atoms can result in one of two chemical reactions: either the breaking a RE−O bond at an anchoring carbonyl or the deprotonation of a hydroxyl group. New N−C bonds are created during the formation of nitro groups, which forms a N−C bond between the N of NO_2_ and an alpha carbon relative to the DOBDC hydroxyl group, Scheme S2.

The formation of new N bonds, specifically N−O bonds, indicate the reactive effects of MOF‐gas interactions within the material. The new functional groups, combined with adsorption of NO_2_ at RE metal sites, provide insight into the possible capture of NO_2_ in competitive gas environments. Such complex reactions could result in degradation of the MOF and require multiple interactions to occur simultaneously.

### HONO formation from NO_2_+H_2_O in the MOF framework

The direct comparison between pure NO_2_ and pure H_2_O in the RE‐DOBDC framework showed the formation of HONO occurs more readily in dry NO_*x*_ gas environments than with humid NO_*x*_. While HONO can form in both dry and humid environments, H_2_O was observed to also interact with DOBDC hydroxyl groups which creates competition at linker sites with NO_2_. The interaction of H_2_O with DOBDC hydroxyl groups can result in H_3_O^+^ formation, Scheme S3, but does not form new bonds with the linker. The effect of humidity is a reduction in the number of interactions NO_2_ can experience with DOBDC hydroxyl H atoms. Even accounting for HONO formation, through proton transfer along a chain of water molecules, does not offset the ability for NO_2_ to deprotonate the DOBDC linker. The spontaneous formation of HONO exemplifies the NO_2_‐MOF interaction found within RE‐DOBDC MOFs. However, formation of HONO in a dry environment is unique, as known mechanisms for HONO formation involve H_2_O.[^34^, ^35^] HONO formation has also been identified in atmospheric mixtures of NO_2_, SO_2_, and H_2_O; this is the only known environment which shows favourable formation of HONO with the inclusion of H_2_O.^[36]^

### Detailed Analysis of AIMD simulations

The commonality found in the HONO formation of RE‐DOBDC MOFs indicates this is a predominant occurrence. The mechanisms of HONO formation are essential to reactive gas species formation in MOFs. This chemical reaction has never been observed experimentally in RE‐DOBDC MOFs and is only isolated through the power of AIMD simulations. Therefore, a deep dive into the simulation results is necessary to understand mechanisms of formation that can be applied in future material design. Below is a detailed accounting of the mechanistic results of AIMD simulations and analyses of (i) HONO formation, (ii) HONO formation conditions, (iii) NO_2_ adsorption in an exemplar RE‐DOBDC MOF (RE=Y), and (iv) the formation of secondary nitrogen species.

### (i) AIMD simulations for a step by step formation timeline of HONO formation

Mechanisms of HONO formation in RE‐DODBC MOFs were found to occur primarily via the binding to a monodentate DOBDC hydroxyl group. Published RE‐DOBDC crystal data identified two linker coordinations, monodentate and bidentate, at 33 % and 67 % in the original unit cell, respectively.^[16]^ The coordination is indicative of native defect sites in MOF materials,[^18^, ^37^] which can be formed due to crystallization kinetics and allows both linker coordinations to exist, Scheme S4. The defect allows for a more flexible coordination within the MOF that can enable metal cluster and linker rotation, known to exist in certain MOFs.[^38^, ^39^] The monodentate linkers also result in an extra hydroxyl group on one third of the organic linkers in RE‐DOBDC MOFs, which serve as reactive sites in the material.

**Scheme 1:**
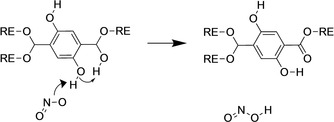
Mechanism of HONO formation via NO_2_ interacting with a monodentate DOBDC hydroxyl group, facilitated by neighbouring hydroxyl.

From the AIMD trajectories, it was observed that as NO_2_ approaches the hydroxyl group of a monodentate linker, a strong hydrogen bonding interaction forms. The H atom of the hydroxyl then begins to transfer between the NO_2_ and the hydroxyl O atom. While all DOBDC linkers have hydroxyl groups, it is the extra neighbouring hydroxyl group found only on monodentate coordination which assists in the formation of HONO. The neighbouring hydroxyl allows a H to be shared between two hydroxyl groups, allowing NO_2_ to take the H and form HONO, Scheme 1.

The transfer of a DOBDC hydroxyl H atom can also occur in two less frequent mechanisms. In the first mechanism, a bidentate coordinated linker can produce HONO following a specific mechanism in which an NO_2_ interacts with the DOBDC hydroxyl group and a second NO_2_ molecule approaches the O to form a nitrate or provide a H atom, Scheme S1. In both cases of H removal from the linker, a secondary interaction is provided to assist the mechanism. In the second mechanism, a H_2_O deprotonates the linker and then transfers the H atom to a nearby NO_2_. The resulting HONO molecular formations include all isomers of the molecule: *trans*‐HONO, *cis*‐HONO, and isonitrous acid (HNO_2_),^[40]^ with the *trans*‐HONO molecule as the primary isomer. The distribution of the HONO isomers is included in Figure S1. The frequent generation of HONO species across the majority of trajectories identifies the strength of interaction in highly competitive environments. It was hypothesized and validated that NO_2_ interacts with the DOBDC linker, but the identification of new HONO products, compared to nitro and nitrate formation, is a new mechanism not formerly identified.

The new species formed from strong NO_2_ interactions are identified for all RE metals simulated. As previously stated, there is no clear trend between strong NO_2_ interactions and the RE metal in the MOF. Given the non‐metal dependent relationship, as well as established similarity in adsorption across the Ln series, the Y‐DOBDC material has been chosen for use in an exemplary trajectory for in depth analysis. The identification and analysis of NO_2_ interactions in Y‐DOBDC are expected to be representative for the same interactions in all RE‐DOBDC MOFs.

### (ii) AIMD analysis of HONO formation conditions

**Figure 4:**
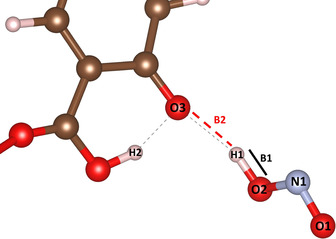
Formation of HONO via interaction of an NO_2_ and monodentate DOBDC linker. C (brown), N (light blue), O (red), and H (white). The bond lengths of O2‐H, B1, and H1‐O3, B2, are compared to determine HONO formation.

The primary HONO formation mechanism is identified to be a NO_2_ molecule deprotonating a monodentate bound DOBDC hydroxyl, as shown in Scheme 1. The atoms and bonds of the NO_2_ and DOBDC hydroxyl are presented in Figure 4.

A HONO molecule containing N1, O1, O2, and H1 is shown interacting with a previously deprotonated DOBDC hydroxyl O atom, O3. As the bond between the NO_2_ and H1 (B1) reaches 1 Å, the HONO molecule is formed. The originally deprotonated O atom (O3) is protonated. This proton comes from a neighbouring hydroxyl group, which is only available on monodentate coordinated DOBDC linkers and identifiable via AIMD.

**Figure 5:**
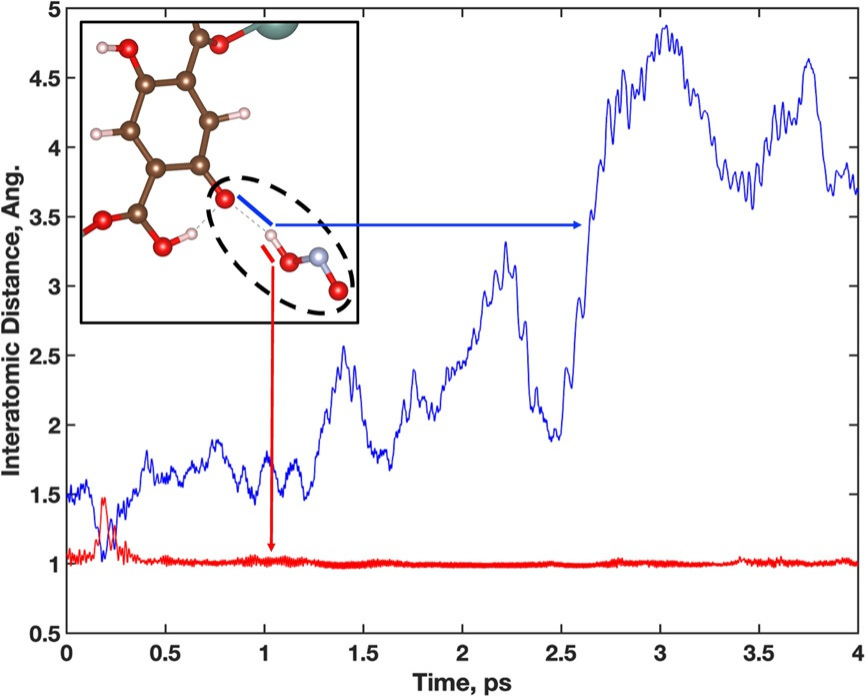
Interatomic distance of H atom between DOBDC hydroxyl group (blue) and NO_2_ molecule (red) during the formation of HONO. H (white), C (brown), O (red), Y (teal), N (light blue).

Determination of when the HONO species forms are identified through calculated interatomic distances, Figure 5. At the beginning of the trajectory, the HONO H atom transfers from the original DOBDC hydroxyl group near time 0.18 ps, as seen in Figure 5. The red and blue H−O bond distances in Figure 5 correspond to the B1 and B2 bonds visualized in Figure 4, respectively. Note that in the snapshot of Figure 4, the deprotonated hydroxyl O (O3) has a strong interaction with a neighbouring DOBDC hydroxyl group. This is possible due to the intrinsic defect in the RE‐DOBDC materials where the anchoring carboxyl O atoms do not all coordinate to metal sites.

After 0.25 ps, the H atom maintains the expected bond distance of ≈1 Å with the NO_2_ molecule (red), completing the formation of the HONO molecule. After the formation of HONO is complete at 0.25 ps, the atomic distances in Figure 5 (blue) corresponds to the relative distance between the HONO molecule and the deprotonated DOBDC hydroxyl group. The calculated distances range between 1.5–4.8 Å as the HONO molecule moves away from the DOBDC. The distances are subsequently used to identify the relationship between interaction energy and distance of the formed HONO molecule. The interaction energy of the HONO molecule within the system was calculated for two time periods along the trajectory from 0–0.5 ps and 2.2–2.8 ps. These two segments of the trajectory capture the energy of the final H transfer between the DOBDC linker and NO_2_ and the HONO molecule dissociating from the deprotonation site.

In the 0–0.5 ps trajectory segment, the HONO molecule is formed both from 0–0.125 ps and again from 0.375–0.5 fs. The average calculated binding energies of HONO with the deprotonated site for these time steps are −0.86 eV and −0.65 eV, respectively. The calculation of time dependent binding energies is defined in the SI. The decreased interaction strength of the HONO with the framework is highly dependent on the distance from the deprotonation site. For example, the average distance from the deprotonated O grows from 1.51 Å in the 0–0.125 ps range to 1.66 Å in the 0.375–0.5 ps range. As the HONO molecule finalizes its dissociation from the deprotonation site in 2.6–2.8 ps, the interaction energy weakens to an average of −0.47 eV. The calculated time dependent binding energy of the HONO molecule shows an increase as the time approaches 2.8 ps. However, this is due to the HONO beginning to interact with a new part of the MOF pore. In using the time dependent interaction energies from the final formation of HONO at 3.75 ps to the final dissociation near 2.8 ps, the change in energy is ≈0.2 eV. Near 0.2 eV the interaction can be classified as physisorption of a HONO molecule with the MOF framework. Otherwise, the average total energy during the final formation of the HONO species changes by −0.2 eV, indicating a stability of the HONO formation.

### (iii) AIMD simulations of NO_2_ adsorption in Y‐DOBDC

The first spontaneous reaction analysed along the Y‐DOBDC + 12 NO_2_ AIMD trajectory is desorption at an unsaturated Y metal site.

**Figure 6:**
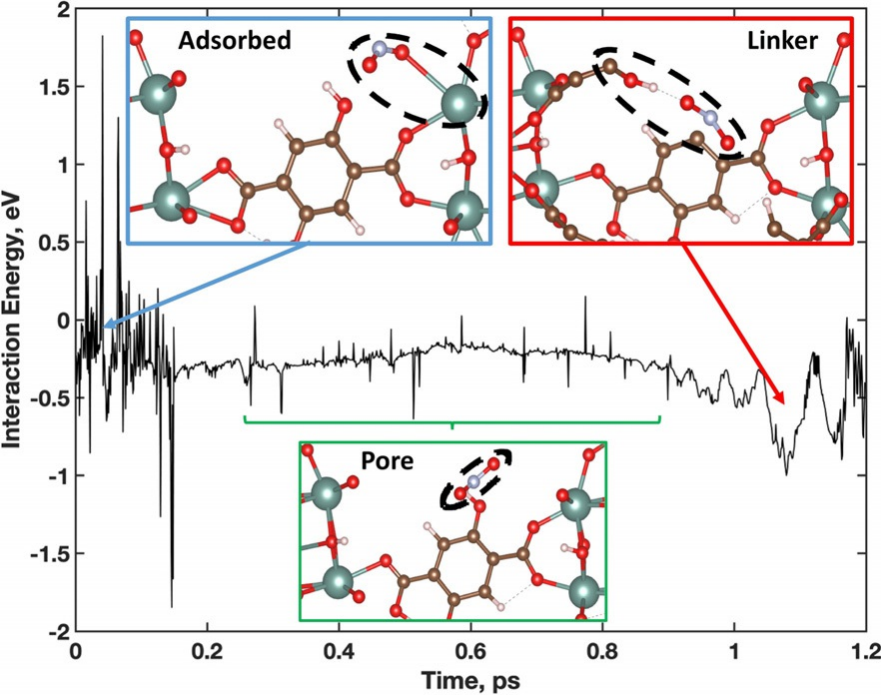
Interaction energy of a NO_2_ adsorbed to Y (blue box), desorbed and free in vacuum (green box), and interacting with a DOBDC linker (red box) along the AIMD trajectory. Inset: H (white), C (brown), O (red), Y (teal), N (light blue).

Examination of a singular NO_2_ molecule that experiences desorption along the AIMD trajectory, Figure 6, shows three phases associated with the MOF‐NO_2_ interaction. First, a NO_2_ molecule is initially adsorbed to an unsaturated metal site (blue box) and then desorbs into free pore space (green box), seen in Figure 6. The calculated interaction energies of each of the NO_2_ locations within the MOF framework is presented in Figure 6, with corresponding snapshots along the AIMD trajectory.

The initial NO_2_ adsorption event shows that the NO_2_ molecule is in physisorption distance of an unsaturated Y metal site; it does not have any secondary interactions with neighbouring linkers or gas molecules. As the NO_2_ desorbs off the MOF framework and into the pore, the interaction energy weakens. It only strengthens again when it a new binding interaction with a DOBDC hydroxyl group. As the NO_2_ approaches the DOBDC linker, the interaction energy again increases to around −1 eV. Interestingly, this interaction energy of NO_2_ with the linker is stronger than the binding energy between NO_2_ adsorbed to the metal site.

**Figure 7:**
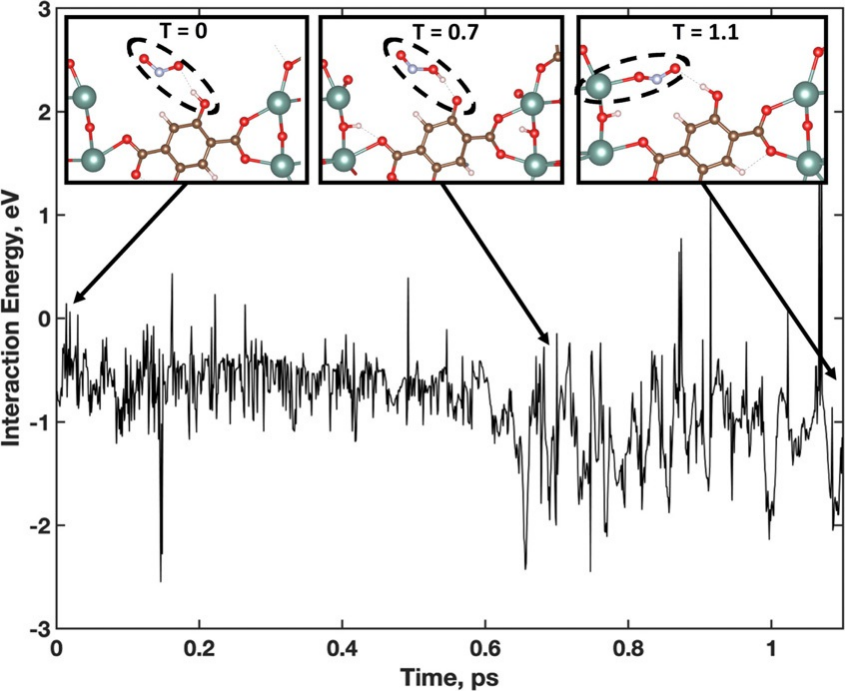
Interaction energy during a NO_2_ adsorption event. Inset snapshots of the interaction show three phases: 1) NO_2_/linker 2) proton passing and HONO formation, 3) adsorption to unsaturated metal site. Inset: H (white), C (brown), O (red), Y (teal), N (light blue).

The inverse of the desorption interaction (adsorption of an NO_2_ molecule to an unsaturated metal site) is also identified along the same trajectory. In this adsorption event, an NO_2_ molecule interacts with a DOBDC hydroxyl group before adsorbing. Inset snapshots along the AIMD trajectory are presented in Figure 7, along with the corresponding interaction energy.

The initial interaction between NO_2_ and the hydroxyl group is shown to be in the same energy region, −0.6 to −1.1 eV, as the previously described NO_2_‐DOBDC interaction. As the NO_2_ nears the DOBDC, a HONO molecule forms 0.7 ps into the interaction with the deprotonation of the DOBDC hydroxyl group. During this interaction, the H atom is transferred back and forth between the NO_2_ and DOBDC. As the DOBDC hydroxyl does not have any neighbouring hydroxyl groups which could pass another proton, the NO_2_ and DOBDC are reformed and the NO_2_ binds at a nearby unsaturated metal site. In this trajectory, the adsorption energy of the NO_2_ is stronger than what was calculated for the desorption event. The additional hydrogen bond formed with the adsorbed NO_2_ strengthens the NO_2_ binding energy.

Prior to permanent NO_2_ adsorption at an unsaturated metal site, the following criteria were met: 1) NO_2_ hydrogen bonded with DOBDC hydroxyl, 2) no neighbouring hydroxyl group were located nearby to donate a proton, 3) NO_2_ was in close proximity to an unsaturated metal, and 4) the continued H‐bond interaction formed with the adsorbed NO_2_. These steps have been identified to achieve sustained adsorption. Other NO_2_ trajectories were observed to show NO_2_‐metal interactions; however, all are short lived and desorbs quickly without any secondary framework interaction.

### (iv) AIMD simulations of the formation of the secondary nitrogen species

**Figure 8:**
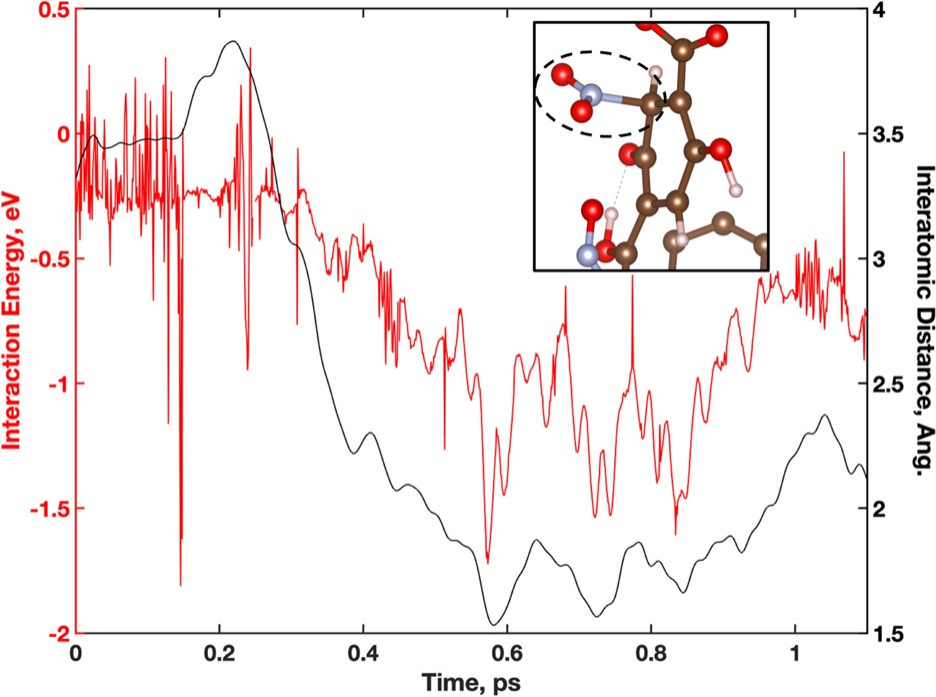
Interaction energy of nitro formation (red) as a function of the C−N bond length (black) along the AIMD trajectory. Inset: H (white), C (brown), O (red), N (light blue).

Species such as nitro, nitrate, and nitrite groups, which form on the DOBDC linkers, were confirmed experimentally by FTIR^[20]^ and have been identified in current AIMD trajectories. One of them, a nitro group (NO_2_) has been further analysed along the Y‐DOBDC + 12 NO_2_ trajectory. Along the first 1 ps of the trajectory, the interaction energy of NO_2_ ^[20]^ has been plotted as with the C−N bond distance, Figure 8.

Initially the NO_2_ molecule is located in the MOF pore and ≈3.5 Å from the nitro‐C atom bond; the average interaction energy of the NO_2_ is approximately −0.15 eV. As the distance of the C−N bond shortens, the interaction energy of the NO_2_ with the C strengthens and reaches a value close to −1.5 eV. This interaction energy agrees with the experimental evidence of NO_2_ preferring NO_2_‐DOBDC interactions as compared to the NO_2_‐Ln metal where the energy of adsorption is close to −0.2 eV. What is also of interest in this interaction is the deprotonated state of the DOBDC linker. As can be seen in the inset geometry of Figure 8. the DOBDC hydroxyl group next to the nitro‐C has been deprotonated via another gas molecule. Importantly, the deprotonation of the neighbouring hydroxyl is not required for nitro formation but was observed in the majority of cases. The unique interaction of NO_2_ binding directly to the MOF DOBDC linkers has previously been identified in M‐MOF‐74.[^11^, ^12^] It was then further confirmed to occur in RE‐DOBDC MOFs.^[20]^ Similar binding was seen in a variety of Zr‐based MOFs, which at times resulted in occurrences of material degradation.^[41]^

## Conclusion

An extensive set of ab initio molecular dynamics (AIMD) simulations were used for the first time to evaluate competitive adsorption of mixed acid gases (NO_2_, H_2_O) in a series of RE‐DOBDC MOFs. The dynamically evolving trajectories allow for evaluation of spontaneous formation of secondary molecular species in the MOF. These simulations account for the effects of confinement by organic linkers and resulting changes in adsorption that cannot be analysed through static density functional theory (DFT) calculations alone.

Analysis of competitive adsorption identified that the gas composition had a stronger influence on gas adsorption than the specific metal centre (Eu, Tb, Y, Yb). Furthermore, adsorbed H_2_O is more frequently bound to metal centres, while NO_2_ can bind with both metal centres and the linker. Additionally, the AIMD trajectory identified the formation of secondary molecular species, including HONO, N_2_O_4_, nitrate groups, nitro groups, and H_3_O^+^.

Of those secondary molecular species formed, the most common is HONO, which is readily formed as a result of deprotonation of the MOF DOBDC organic linker, and not from the interaction between NO_2_ and H_2_O in the pore space. This is a previously unreported mechanism of HONO formation in MOFs that was discovered, and is reported herein, through this unique application of AIMD methodologies. The unique DOBDC coordination found specifically in RE‐DOBDC MOFs allows for proton transfer from the linker to the adsorbed NO_2_ without the presence of H_2_O. Additionally, several proton transfer events occur prior to HONO formation.

Through the combination of AIMD simulations and validation from previous experiment, unprecedent insight into mechanisms of acid gas separation can be developed not only in MOFs but across porous organic systems. The AIMD simulations provide step‐wise improvement in accuracy of computational results including temperature effects and spontaneous reactions. Identification of individual strong MOF‐gas interactions in RE‐DOBDC MOFs provides fundamental understanding of the separation capability and maintained stability of RE‐MOFs under acid gas conditions. Further application of AIMD to simulated mixed acid gas adsorption in MOFs will allow for identification of mechanisms that control competitive adsorption processes with even more complex gas streams. This will also enable future computational design of materials around these adsorption mechanisms.

## Conflict of interest

The authors declare no conflict of interest.

## Supporting information

As a service to our authors and readers, this journal provides supporting information supplied by the authors. Such materials are peer reviewed and may be re‐organized for online delivery, but are not copy‐edited or typeset. Technical support issues arising from supporting information (other than missing files) should be addressed to the authors.

Supplementary
