# Supplementary material for: Prediction of Reactive Nitrous Acid Formation in Rare‐Earth MOFs via ab initio Molecular Dynamics
Source: Angew Chem Int Ed Engl. 2021 Apr 8;60(20):11514–22. doi: 10.1002/anie.202102956 (PMC8252009; doi:10.1002/anie.202102956)
Supplement: Supplementary file 1 — Supplementary [file ANIE-60-11514-s001.pdf]

Angewandte Chemie
Eine Zeitschrift der Gesellschaft Deutscher Chemiker

Supporting Information

Prediction of Reactive Nitrous Acid Formation in Rare-Earth MOFs via ab initio Molecular Dynamics

Dayton J. Vogel, Jessica M. Rimsza, and Tina M. Nenoff**

anie_202102956_sm_miscellaneous_information.pdf

Supporting Information
Table of Contents

Experimental Procedures

Computational Methodology – Page 2

Results and Discussion

Table S1 – Page 3

Table S2 – Page 3

Table S3 – Page 3

Scheme S1 – Page 3

Scheme S2 – Page 4

Scheme S3 – Page 4

Figure S1 – Page 4

References – Page 4

Computational Methodology

Periodic bulk calculations, ground state electronic structures, and geometries were optimized for RE-DOBDC MOFs (RE=Y, Eu, Tb, Yb) using spin unrestricted DFT as implemented in the Vienna Ab initio Simulation Package (VASP) code[1],[2] in a plane wave basis set,[3],[4] with projector-augmented wave (PAW) potentials.[5],[6] Large core potentials (LCPs), which represent the RE(III) oxidation state, with a 600 eV cutoff energy were used and converged to a force accuracy of 0.03 eV per atom with gamma point for k-point sampling. A Gaussian smearing of 0.01 eV was used for smearing of the electron occupation. The generalized gradient approximation (GGA) exchange correlation functional of Perdew, Burke, and Ernzerhof designed for solids and surfaces (PBEsol)[7] is chosen for consistency with recent successes in RE-MOFs. The DFT-D3 method of Grimme with Becke–Jonson damping is added as a van der Waals correction.[8] The following computational procedure was used as a reproduceable process for optimizing RE-DOBDC MOF structural geometries when interchanging RE elements into the framework. The experimental structure for Eu-DOBDC MOF was used as the initial MOF structure and has been experimentally reported from x-ray diffraction analysis.[9] The Eu-DOBDC model consists of a $Eu_{12}(\mu_3\text{-}OH)_{16}(C_8O_6H_4)_8(C_8O_6H_5)_4$ unit cell with a tetragonal structure, P4/mnc. To visualize the MOF structure, a snapshot of the Y-DOBDC + 12 NO_2 is presented in Figure 1.

The structural relaxation of RE-DOBDC MOFs consists of four steps: the replacement of Eu with the RE metal of interest, optimization of atomic positions, optimization of atomic position, cell shape, and cell volume, and re-optimization of atomic positions. Following final geometry optimization, a distribution of H_2O, NO_2, and SO_2 gas molecules were placed randomly in the unit cell via Packmol.[10] The MOF + gas systems again had the atomic positions optimized to minimize the total energy. The number of gas molecules placed into the unit cell pores for each simulation, in Table 1. The gas mixtures include single gas molecules (H_2O, NO_2, SO_2) as a control for a single gas molecule in a pore. Higher concentration single component gas mixtures (12 H_2O, 12 NO_2, 12 SO_2) were simulated to increase the number of interactions between the single component gases and the framework and to mimic a more realistic pore filling condition. A maximum of 12 acid gas molecules were simulated as it can reach 100% metal site adsorption as there are 12 metal atoms in the unit cell. Following single component mixtures, binary gas mixtures (H_2O:NO_2, H_2O:SO_2, NO_2:SO_2) were simulated, including at low (1:1 ratio) and high (6:6 ratio) concentration. Finally, ternary acid gas mixtures (H_2O:NO_2:SO_2) were simulated, again at low (1:1:1) and high (4:4:4) concentration.

AIMD simulations were started from the optimized MOF + gas models and maintained the same input parameters as for the initial optimizations. The AIMD trajectories were calculated in two parts. A microcanonical ensemble (NVE) was performed to bring the model to ambient temperature. The velocities were rescaled every 10 steps until the temperature stabilized at 300K ± 20K. The rescaling was complete following 600 steps with a time step of 0.5 fs. Second, a longer NVE calculation was performed using the scaled velocities as initial input. The longer production NVE trajectories were calculated for 10 ps, using a 1fs timestep. All trajectories were analyzed at each time step for various gas-MOF and gas-gas interactions and recorded. The calculated binding energies along the AIMD trajectory used the same parameters as listed above for optimization. The geometries were extracted at each time step along the AIMD calculation and used to calculate single point energy values required for binding energy:

$E_b(t) = E_{MOF+Gas}(t) - E_{MOF}(t) - E_{Gas}(t)$

Results and Discussion

Table S1. New chemical species identified along AIMD trajectories of RE-DOBDC MOFs.

RE Metal	Gas Mixture					
	H_2O	12 H_2O	NO_2	12 NO_2	NO_2 + H_2O	6NO_2 + 6H_2O
Eu	-	H_3O^+	HONO	HONO, N_2O_4, nitrate, nitro	HONO	HONO, N_2O_4
Tb	-	H_3O^+	HONO	HONO, N_2O_4, nitro	HONO, H_3O^+	HONO, H_3O^+
Y	-	H_3O^+	HONO	HONO, N_2O_4, nitro	HONO, H_3O^+	HONO, H_3O^+
Yb	-	H_3O^+	HONO	HONO, nitro	HONO, H_3O^+	HONO

Table S2. Acid gas species (number) adsorbed at metal sites in all AIMD trajectories.

RE Metal	Gas Mixture					
	H_2O	12 H_2O	NO_2	12 NO_2	NO_2 + H_2O	6NO_2 + 6H_2O
Eu	-	H_2O (5)	-	NO_2 (1)	-	NO_2(1), H_2O (2)
Tb	-	H_2O (2)	-	HNO_2 (1)	-	H_2O (1)
Y	H_2O(1)	H_2O (2)	-	NO_2 (1)	-	NO_2 (1), H_2O (1)
Yb	-	H_2O (1)	-	NO_2 (1)	-	H_2O (2)

Desorption: Eu(6:6 NO_2 (1)); Tb(6:6 NO_2 (1)); Y(12 H_2O(1)); Y(6:6 NO_2 (1),H_2O(1))

Table S3. Unit cell volumes of RE-DOBDC MOFs calculated.

RE Metal	Volume (Å^3)
Eu	5143.9
Tb	5047.3
Y	4945.5
Yb	4851.4

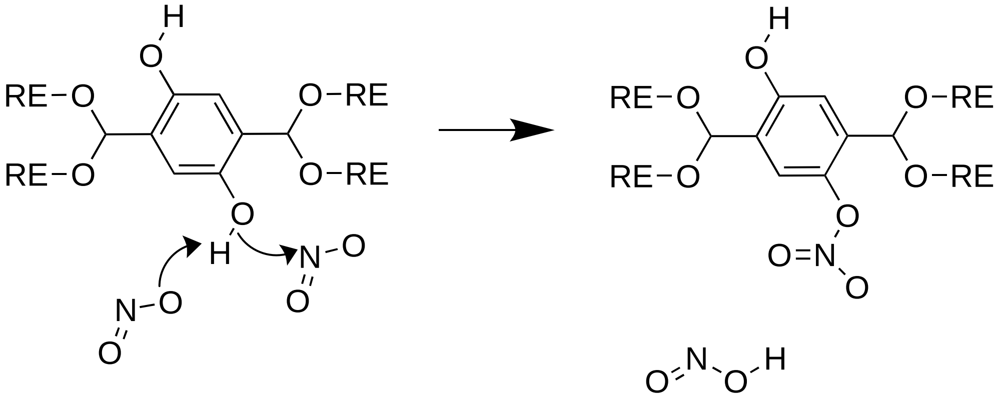

Scheme S1. The formation of nitrate groups identified along AIMD trajectories occur via deprotonation of a DOBDC hydroxyl group, providing a location for a NO_2 molecule to bind.

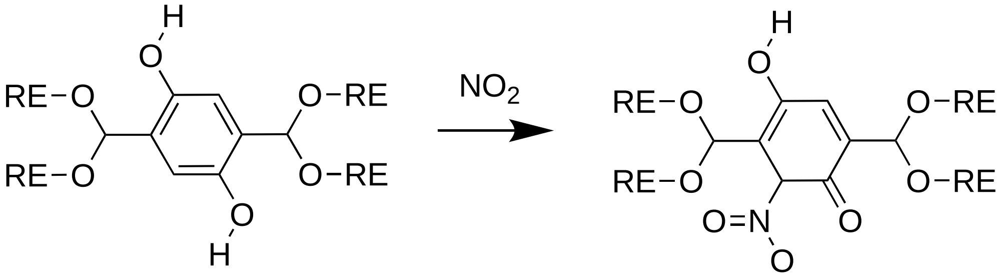

Scheme S2. Nitro group formation identified along AIMD trajectories occurs when NO2 binds at an alpha carbon next to a DOBDC hydroxyl group.

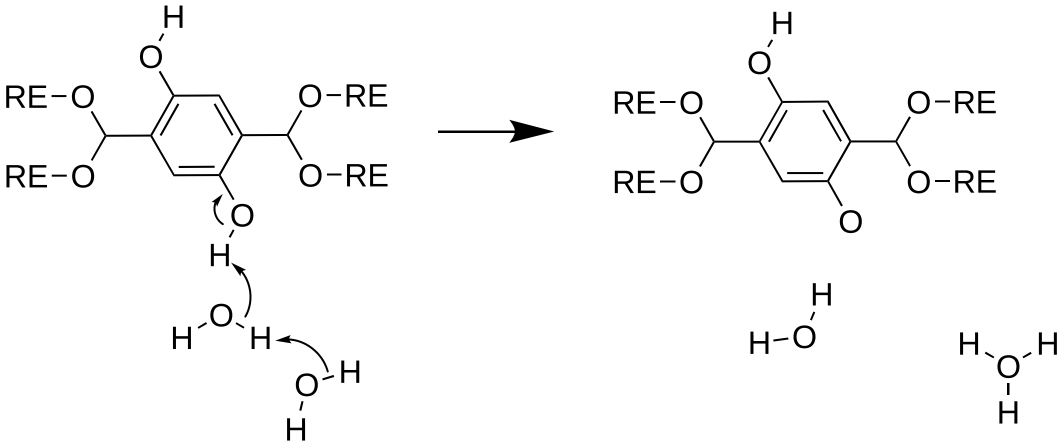

Scheme S3. Hydronium formation facilitated via proton passing with neighboring H_2O molecule.

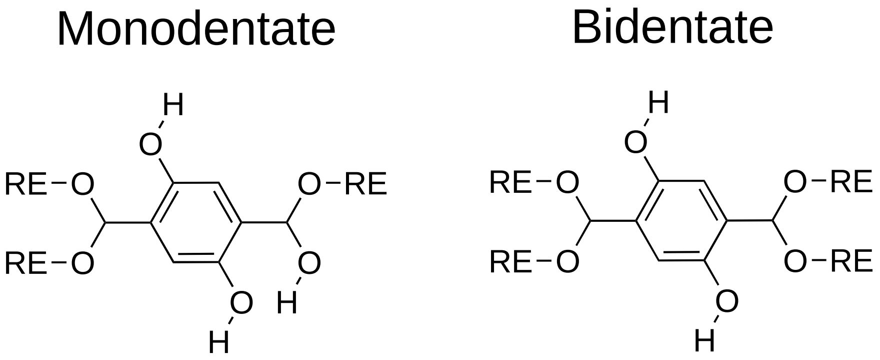

Figure S1. Monodentate and bidentate coordination of DOBDC linkers in RE-DOBDC MOFs. The monodentate linker has three carboxylic-RE bonds compare to four in the bidentate coordination.

References

[1] G. Kresse, J. Hafner, *Phys. Rev. B* **1993**, *47*, 558-561.
[2] G. Kresse, J. Hafner, *Phys. Rev. B* **1994**, *49*, 14251-14269.
[3] G. Kresse, J. Furthmüller, *Comput. Mater. Sci.* **1996**, *6*, 15-50.
[4] G. Kresse, J. Furthmüller, *Phys. Rev. B* **1996**, *54*, 11169-11186.
[5] P. E. Blöchl, *Phys. Rev. B* **1994**, *50*, 17953-17979.
[6] G. Kresse, D. Joubert, *Phys. Rev. B* **1999**, *59*, 1758-1775.
[7] J. P. Perdew, A. Ruzsinszky, G. I. Csonka, O. A. Vydrov, G. E. Scuseria, L. A. Constantin, X. Zhou, K. Burke, *Phys. Rev. Lett.* **2008**, *100*, 136406.
[8] S. Grimme, S. Ehrlich, L. Goerigk, *J. Comput. Chem.* **2011**, *32*, 1456-1465.
[9] D. F. Sava Gallis, L. E. S. Rohwer, M. A. Rodriguez, M. C. Barnhart-Dailey, K. S. Butler, T. S. Luk, J. A. Timlin, K. W. Chapman, *ACS Appl. Mater. Interfaces* **2017**, *9*, 22268-22277.
[10] L. Martínez, R. Andrade, E. G. Birgin, J. M. Martínez, *J. Comput. Chem.* **2009**, *30*, 2157-2164.
